# Development of the infant gut microbiome predicts temperament across the first year of life

**DOI:** 10.1017/S0954579421000456

**Published:** 2021-06-10

**Authors:** Molly Fox, S. Melanie Lee, Kyle S. Wiley, Venu Lagishetty, Curt A. Sandman, Jonathan P. Jacobs, Laura M. Glynn

**Affiliations:** 1 Department of Anthropology, UCLA, Los Angeles, CA, USA; 2 Department of Psychiatry & Biobehavioral Sciences, UCLA, Los Angeles, CA, USA; 3 Division of Gastroenterology, Hepatology and Parenteral Nutrition, VA Greater Los Angeles Healthcare System, Los Angeles, CA, USA; 4 The Vatche and Tamar Manoukian Division of Digestive Diseases, Department of Medicine, David Geffen School of Medicine at UCLA, Los Angeles, CA, USA; 5 UCLA Microbiome Center, David Geffen School of Medicine at UCLA, Los Angeles, CA, USA; 6 Department of Psychiatry and Human Behavior, UC Irvine, Irvine, CA, USA; 7 Department of Psychology, Chapman University, Orange, CA, USA

**Keywords:** emotion regulation, gut–brain axis, infant development, microbiome, temperament

## Abstract

Perturbations to the gut microbiome are implicated in altered neurodevelopmental trajectories that may shape life span risk for emotion dysregulation and affective disorders. However, the sensitive periods during which the microbiome may influence neurodevelopment remain understudied. We investigated relationships between gut microbiome composition across infancy and temperament at 12 months of age. In 67 infants, we examined if gut microbiome composition assessed at 1–3 weeks, 2, 6, and 12 months of age was associated with temperament at age 12 months. Stool samples were sequenced using the 16S Illumina MiSeq platform. Temperament was assessed using the Infant Behavior Questionnaire-Revised (IBQ-R). Beta diversity at age 1–3 weeks was associated with surgency/extraversion at age 12 months. *Bifidobacterium* and *Lachnospiraceae* abundance at 1–3 weeks of age was positively associated with surgency/extraversion at age 12 months. *Klebsiella* abundance at 1–3 weeks was negatively associated with surgency/extraversion at 12 months. Concurrent composition was associated with negative affectivity at 12 months, including a positive association with *Ruminococcus-1* and a negative association with *Lactobacillus*. Our findings support a relationship between gut microbiome composition and infant temperament. While exploratory due to the small sample size, these results point to early and late infancy as sensitive periods during which the gut microbiome may exert effects on neurodevelopment.

## Background

The human gastrointestinal tract is home to trillions of microbial cells that make up an ecosystem that is increasingly recognized as a central component of human physiology, health, and wellbeing (Bordenstein & Theis, 2015; Human Microbiome Project Consortium, 2012; Peterson et al., 2009). The early postnatal phase of the human life span is a period of remarkable plasticity, characterized by dynamic interplay between extrinsic and intrinsic ecology and systems. The first year of life is characterized by sensitive periods of development for both the brain and gut (Borre et al., 2014; Jašarević, Morrison, & Bale, 2016; Stilling, Dinan, & Cryan, 2014)’ and brain maturation coincides with pioneer microbial colonization of the gastrointestinal tract. The early life window of sensitivity of these biological systems is followed by long-term stable phenotypes, thus rendering the first year of life a period during which critical developmental progress occurs (Charbonneau et al., 2016; Hensch, 2004). Overlapping sensitive periods of these biologically interconnected systems suggests that maturation of the gut microbiome may have lifelong consequences for neuropsychological function. Preclinical studies support this hypothesis and indicate that dysbiosis in the gut during sensitive periods of early life development may detrimentally impact neurodevelopment in ways that shape emotion regulation and affective disorder risk across the life course (Rogers et al., 2016; Sampson & Mazmanian, 2015). However, few studies have investigated associations between the microbiome and neurodevelopment of humans.

### Overlapping sensitive periods of gut and brain development

The first year of life is a sensitive window for development, involving extraordinary plasticity for both the gut and the brain. Developmental plasticity describes the ability of an individual to produce a range of phenotypes depending on the conditions and exposures encountered during development (Gluckman, Cutfield, Hofman, & Hanson, 2005). Humans are sensitive to a variety of environmental cues that may shape the trajectory of phenotypic specification (Gluckman, Hanson, Spencer, & Bateson, 2005). Plasticity is not unlimited and individuals tend to exhibit less plasticity with age due to irreversible canalization (Bateson, 2001). Infancy is a period of heightened neuroplasticity as it is characterized by rapid brain growth (Knickmeyer et al., 2008; Tau & Peterson, 2010), massive outgrowth of dendrites and axons, and synaptogenesis alongside synaptic pruning (Huttenlocher & Dabholkar, 1997; Petanjek, Judas, Kostović, & Uylings, 2008). Glial cells proliferate in the subventricular zone of the forebrain, migrate across brain regions, and differentiate into oligodendrocytes and astrocytes (Menn et al., 2006; Sharon, Sampson, Geschwind, & Mazmanian, 2016), which facilitates synaptic pruning by complement activation and phagocytosis (Hong & Stevens, 2016).

Coincident with neurodevelopmental progress, human infants exhibit acquisition of cognitive abilities and emotion regulation capabilities during the first year. The development of temperament is a critical aspect of self-regulation that emerges in this period, and infant temperament development is a plastic process influenced by several biological factors (Fox, Henderson, Pérez-Edgar, & White, 2008). However, the specific biological factors that shape inter-individual differences remain unclear. While modest changes in temperament may reflect maturational changes across development (Montroy, Bowles, Skibbe, McClelland, & Morrison, 2016), it is considered relatively stable after childhood and features of early temperament have been shown to predict personality and adverse mental health outcomes later in life (Laceulle, Ormel, Vollebergh, van Aken, & Nederhof, 2014; Rothbart & Posner, 2015; Sayal, Heron, Maughan, Rowe, & Ramchandani, 2014). Negative affectivity and emotional reactivity, especially, are associated with risk of behavioral and emotional problems in childhood (Abulizi, Pryor, Michel, Melchior, & van der Waerden, 2017), as well as later depressive and anxiety symptoms (Compas, Connor-Smith, & Jaser, 2004; De Pauw & Mervielde, 2010), attention-deficit/hyperactivity disorder (Sullivan et al., 2015), and autism (Clifford, Hudry, Elsabbagh, Charman, & Johnson, 2013).

The first year is also a sensitive window for gut microbiome development. From birth to age 1 year, the gut microbiome undergoes significant changes, resulting in a community structure that is much more stable after 1 year of age and virtually matured to adult status by 3 years of age (Yatsunenko et al., 2012). The first few days of life set the developmental trajectory of the gut microbiome as pioneer species of *Escherichia, Staphylococcus*, and *Streptococcus* typically dominate and produce anaerobic environments (Pantoja-Feliciano et al., 2013), inviting subsequent growth of anaerobic genera such as *Bifidobacterium* and *Bacteroides* (Cong, Henderson, Graf, & McGrath, 2015). During the first few weeks of life, the gut typically comes to be dominated by *Bacteroides, Bifidobacterium, Parabacteroides, Escherichia*, and *Shigella* (Bäckhed et al., 2015). Specifically, newborn gut microbial colonization begins with microbial communities from the mother’s birth canal ingested during parturition (Dominguez-Bello, Blaser, Ley, & Knight, 2011; Jašarević et al., 2016; Jašarević, Rodgers, & Bale, 2015; Mueller, Bakacs, Combellick, Grigoryan, & Dominguez-Bello, 2015; Pantoja-Feliciano et al., 2013; Song, Dominguez-Bello, & Knight, 2013), followed by breast milk (Le Doare, Holder, Bassett, & Pannaraj, 2018), and subsequent exposure to extrinsic environmental exposures (Biasucci et al., 2010; Dominguez-Bello et al., 2010; Palmer, Bik, DiGiulio, Relman, & Brown, 2007). Within population-level trends, there is individual-level variation in timing and community structure (Bäckhed et al., 2015; Sharon et al., 2016; Yassour et al., 2016). The colonization of the gut microbiome also exhibits phenotypic variability as human infants present different microbial composition across environmental and cultural contexts (Sprockett et al., 2020).

### Gut microbiota and neurodevelopment

Many of the phenotypes that affect health are connected to the gastrointestinal system (the gut controls energy availability and coordinates metabolic processes that fuel central and somatic functions) and the central nervous system. Furthermore, the gut and brain are physiologically connected in a bidirectional communication and control system. Multiple pathways link the gut microbiome and brain, including vagal nerve innervation, microbial production of neuromodulatory metabolites, and alterations to innate immunity (Jašarević et al., 2015). The vagus nerve is a two-way neural connection between the gut and brain, with a sensitive period of development of enteric axon terminals occurring early in postnatal life (Ratcliffe, Farrar, & Fox, 2011) – a process that could plausibly be influenced by gut microbiota (Sommer & Bäckhed, 2013). In this way, the “microbiome–gut–brain axis” is fundamentally implicated in biological regulation of health.

While it has been speculated that dysbiosis in the gut during sensitive periods of early life development may detrimentally impact neurodevelopment (Diaz Heijtz, 2016; O’Mahony, Clarke, Dinan, & Cryan, 2017), few studies have examined the change in gut microbiome across infancy in humans. One prospective longitudinal study that included multiple assessments of microbiome across infancy found that alpha diversity at 12 months of age was negatively associated with neurodevelopment (cognitive and language scores) at age 2 years (Carlson et al., 2018). A longitudinal study of 201 children with fecal microbiome composition data at 1, 6, and 12 months of age found that an abundance of *Prevotella* was inversely associated with increased internalizing symptoms at age 2 years (Loughman, Ponsonby, et al., 2020).

To our knowledge, only two studies have investigated associations between composition of the gut microbiome and temperament in human infants, and one study in toddlers. Wang et al. (2020) found that an abundance of the genus *Bifidobacterium* was positively associated with soothability and a relative abundance of *Hungatella* was negatively associated with cuddliness in 12-month-old infants. In a subcohort from the FinnBrain Birth Cohort Study, Aatsinki et al. (2019) found that higher abundances of *Bifidobacterium* and *Streptococcus* and a lower abundance of *Atopobium* at age 2.5 months were associated with greater surgency/extraversion scores, measured using the Infant Behavior Questionnaire-Revised (IBQ-R), in 6-month-old infants. These results were sex-specific, with only boys showing associations between *Bifidobacterium* and surgency. The third study investigated associations between the gut microbiome and temperament in toddlers aged 18–27 months (Christian et al., 2015). It found that gut microbial phylogenetic diversity was positively associated with surgency/extraversion.

While these limited studies support associations between gut microbiome composition and neurodevelopment in infancy, more longitudinal studies are needed to examine the role of the microbiome in the development of infant temperament. It is also necessary to examine prospective effects at earlier ages than previously have been considered, given the dramatic changes that occur in gut microbial composition across the first 2 months of life.

### This study

The aim of this exploratory study was to investigate associations between gut microbiome composition across the first year of life and infant temperament. We took a longitudinal approach and quantified associations between gut microbiome composition (diversity and genera) at ages 1–3 weeks, 2, 6, and 12 months and temperament at 12 months of age. This is the first study, to our knowledge, to investigate associations between composition of the gut microbiome as early as 1–3 weeks of age (or any timepoint earlier than 2.5 months of age) and temperament later in infancy. We conducted exploratory analyses with three validated dimensions of temperament. When a dimension exhibited a significant relationship with microbiome composition, we conducted a follow-up exploration of how each component subscale for that domain related to microbiome composition in order to check whether a particular subcomponent was driving the trend. Following the extant literature, we hypothesized that microbiome composition would be associated with variation in temperament in the dimensions of surgency/extraversion and negative affectivity This study sheds light on the biological mechanisms that influence inter-individual differences in temperament.

## Method

### Cohort

This project utilized data from a larger, prospective, longitudinal cohort study of mother–child dyads in Southern California, the Pregnancy Experiences and Infant Development Study (PEIDS) (P50/MH096889). Women were offered voluntary participation in PEIDS, recruited through their clinicians’ offices, email, and print announcements. Written informed consent was obtained from mothers for their own and their infants’ participation after full study procedures were described. PEIDS and our microbiome substudy were approved by the institutional review boards of participating institutions. Our study adhered to the tenets of the Declaration of Helsinki. We capitalized on PEIDS data collection in which visits occurred every few weeks and involved mother–child psychological, behavioral, cognitive, and biomedical assessments. This study included a subset of the cohort and involved four sessions: a home visit 1–3 weeks after birth and sessions at a clinical research site when the child was aged 2, 6, and 12 months. This subset comprised infants who produced stool during these sessions. Therefore, participants were not preselected or actively selected for the substudy because it was based on the random occurrence of when the infant produced stool.

### Sample collection and processing

Visit protocols involved ~2.5 hr of assessments, both related and unrelated to the current project. When the infant produced stool, the diaper was collected by study staff, who covered the stool with film to seal the sample during transport. For home visits, the entire diaper was then sealed in a plastic bag and transported in a hard-sided cooler to the laboratory (maximum 45 min). Visits at age 2, 6, and 12 months occurred at a clinical site with a laboratory. The study staff transferred stool into OMNIgene gut collection kits (OMR-200, DNA Genotek), aliquoted the mixture into cryovials, and stored them at −80°C.

### Infant temperament

To assess infant temperament, mothers completed the IBQ-R when the infant was 12 months old (Gartstein & Rothbart, 2003). The IBQ-R includes 191 questions addressing concrete behaviors; for example, “During a peek-a-boo game, how often did the baby smile” and “How often during the last week did the baby startle to a sudden or loud noise.” The IBQ-R was developed to reduce the possibility of maternal reporting bias by asking about specific behaviors in defined situations, rather than asking for judgments about child temperament or behaviors. Responses on these scales range from 1 = *never* to 7 = *always*. The IBQ-R measures three broad dimensions of temperament: negative affectivity, surgency/extraversion, and orienting/regulation. The negative affectivity dimension is created by averaging scores across four subscales assessing sadness, fear, falling reactivity, and distress to limitations. The surgency/extraversion dimension consists of six subscales assessing approach, vocal reactivity, high-intensity pleasure, smiling/laughter, activity level, and perceptual sensitivity. The orienting/regulation dimension comprises the subscales cuddliness/affiliation, low-intensity pleasure, duration of orienting, and soothability. This widely used parental-report instrument exhibits good internal reliability and validity (Goldsmith & Campos, 1990; Worobey & Blajda, 1989), and correlates well with infant behavioral observations (Worobey & Blajda, 1989). In our cohort, IBQ-R dimensions had Cronbach’s alphas as follows: negative affectivity, α = .77; surgency/extraversion, α = .95; orienting/regulation, α = .89; subscales mean α = .80, standard deviation (*SD*) = .04.

### 16S ribosomal RNA gene sequencing

Stool samples were submitted to DNA Genotek for DNA extraction and sequencing of the V3–V4 region of the 16S ribosomal RNA gene by Illumina MiSeq v3 according to a published protocol (Klindworth et al., 2013). DADA2 was used to perform quality filtering, merge paired end reads, remove chimeras, and cluster sequences into exact amplicon sequence variants (Callahan et al., 2016). Forward reads were truncated to 280 base pairs and reverse reads to 220 base pairs. Reads were removed if the expected error rate exceeded two base pairs or if a single nucleotide had a Phred score of two or less. After these processing steps, the sequence depth ranged from 6,958 to 72,100 with a mean of 30,398. Taxonomy was assigned for amplicon sequence variants based on the SILVA database down to the level of family, genus, or species, depending on the depth of reliable classifier assignments (Quast et al., 2013).

### Bioinformatics and statistical methods

Significance in differential mean relative abundances of each phylum, family, and genus across timepoints was determined using Kruskal–Wallis test. Microbial alpha diversity was assessed on data sets rarefied to equal sequencing depth (6,958) using the Chaol index (richness) and Shannon index (evenness and richness). Significance for differences in alpha diversity measures by age was determined using one-way analysis of variance (ANOVA) adjusting for subject to control for effects of repeated sampling from the same individual. Beta diversity of the unrarefied genus-level data set after removing genera that were present in less than 10% of the samples was calculated using robust Aitchison distances implemented with the DEICODE plugin in QIIME 2, then visualized with principal coordinates analysis (Martino et al., 2019). The significance of differences in beta diversity was assessed using permutational multivariate analysis of variance (PERMANOVA). Significance testing for changes in relative abundance at phylum level by age was performed by one-way ANOVA, adjusting for subject. The differential abundance of microbial genera present in at least 10% of the samples was determined using multivariate negative binomial mixed models in DESeq2 (Love, Huber, & Anders, 2014). The unrarefied genus counts were normalized by the size factor (median value of all ratios for a given sample). Results of differential abundance testing were adjusted for multiple hypotheses testing with a significance threshold of false discovery rate <0.1.

For the multivariate models, a two-step process was implemented to decide which covariates to include. First, a list of potential covariates was identified based on previous literature indicating the possibility of relationships with both infant temperament and microbiome composition, or otherwise justified by previous literature. To this end, infant sex (Gartstein & Rothbart, 2003; Martin et al., 2000), breastfeeding duration (Ho et al., 2018; Rogers & Blissett, 2019; Wasser et al., 2011), and antibiotic use (Kim, Sitarik, Woodcroft, Johnson, & Zoratti, 2019) were selected as covariates based on previous studies. Breastfeeding duration included both exclusive and mixed feeding, as long as breastmilk was being given. The mode of delivery was also included because all three previous studies of the relationship between early life temperament and gut microbiome utilized this control variable (Aatsinki et al., 2019; Carlson et al., 2018; Loughman, Ponsonby, et al., 2020). It should also be noted that mode of delivery appears to influence brain (Castillo-Ruiz, Mosley, Jacobs, Hoffiz, & Forger, 2018), gut microbial composition, and social behavior (Morais et al., 2020) in animal models.

Second, each variable was tested in a univariable model predicting beta diversity outcomes using the age-based subsets or the entire data set after adjusting for age (Supplementary Material Table S1). Selection criteria for covariates included at least one significant (*p* < .05) association with microbial beta diversity in any subgroup. We did not adjust for variables that had no statistical relationship with microbiome composition at any timepoint. Consequently, we adjusted the multivariate models for infant sex and breastfeeding duration, and not mode of delivery or antibiotic use.

Our statistical methods involved the following analyses. (a) We assessed alpha diversity differences by age as measured by the Chao1 index (species richness) and the Shannon index (evenness and richness) by ANOVA, adjusting for subject. (b) We assessed beta diversity differences by age using PERMANOVA, adjusting for subject, and visualized the effect using principal coordinates analysis plots of the microbial beta diversity measured by DEICODE distances. (c) We compared the mean relative abundances in each age group of bacterial clades at phylum, family, and genus levels. (d) We assessed the association between alpha diversity at each timepoint and IBQ-R scores at 12 months of age using multivariate linear regression models, adjusting for infant sex and breastfeeding duration. (e) We assessed the association between beta diversity at each timepoint and IBQ-R scores at 12 months of age using PERMANOVA, adjusting for infant sex and breastfeeding duration. (f) For significant associations encountered in step (e). we conducted differential abundance testing to identify specific genera associated with the corresponding IBQ-R scores at 12 months of age.

## Results

### Gut microbial diversity and composition change across infant age groups

**Figure 1.**
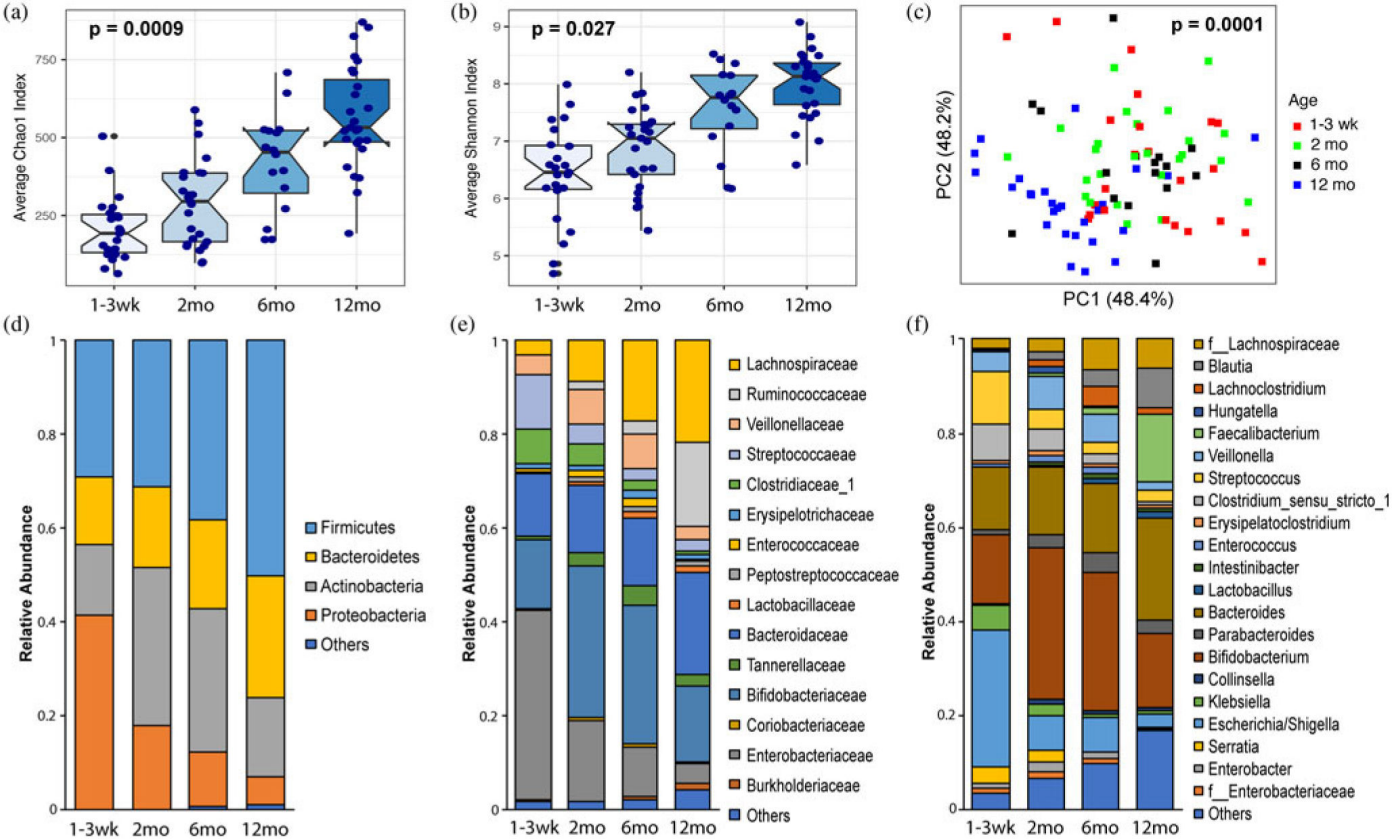
Changes in infant fecal microbiota at different ages during the first year of life representing 91 samples collected from 67 infant donors. (a) and (b) Notched boxplots of alpha diversity as measured by (a) Chao1 index (species richness) and (b) Shannon index (evenness and richness). *P* value for alpha diversity differences by age was determined by one-way ANOVA (analysis of variance) after adjusting for subject. The 95% confidence interval around the median is displayed by the notch. (c) Principal coordinates analysis plots of microbial beta diversity measured by DEICODE distances. Each symbol represents a sample that is colored by age at the time of sample collection. *P* value for beta diversity differences by age was calculated using PERMANOVA (permutational multivariate analysis of variance) after adjusting for subject. (d)–(f) Stacked bar charts showing mean relative abundance in each age group of bacterial (d) phyla, (e) families, and (f) genera. Others indicate sum of taxa present at less than 2% in mean relative abundance averaged over different age groups.

In total, 91 samples were collected at different ages (1–3 weeks, 2, 6, and 12 months) from 67 infant donors (Table 1). Similar to findings from other infant microbiome studies (Hill et al., 2017; Niu et al., 2020), we found a significant increase in alpha diversity with age according to the Chao1 (*p* < .001) and Shannon (*p* = .027) indices (Figures 1a and 1b). Beta diversity analysis demonstrated significant microbial community alterations by age after adjusting for participant to account for inter-individual differences (Figure 1c; *p* < .001). At the phylum level, the mean relative abundance of *Firmicutes* increased (*p* = .005) with age and the mean relative abundance of *Proteobacteria* decreased (*p* = .004) with age after adjusting for the participant (Figure 1d; Supplementary Material Figure S1). Changes in *Firmicutes* were largely driven by increases in genera *Blautia* and *Faecalibacterium* with age, which belong to families *Lachnospiraceae* and *Ruminococcaceae*, respectively. Changes in *Proteobacteria* were driven by decreases in genera *Klebsiella, Escherichia/Shigella*, and *Serratia*, all of which belong to the family *Enterobacteriaceae*. Genera *Bacteroides* (13%–22%) and *Bifidobacterium* (15%–32%), belonging to phyla *Bacteroidetes* and *Actinobacteria*, respectively, were present in abundance throughout the first year of life (Figures 1d-1f).

### Gut microbiota composition associations with temperament

**Figure 2.**
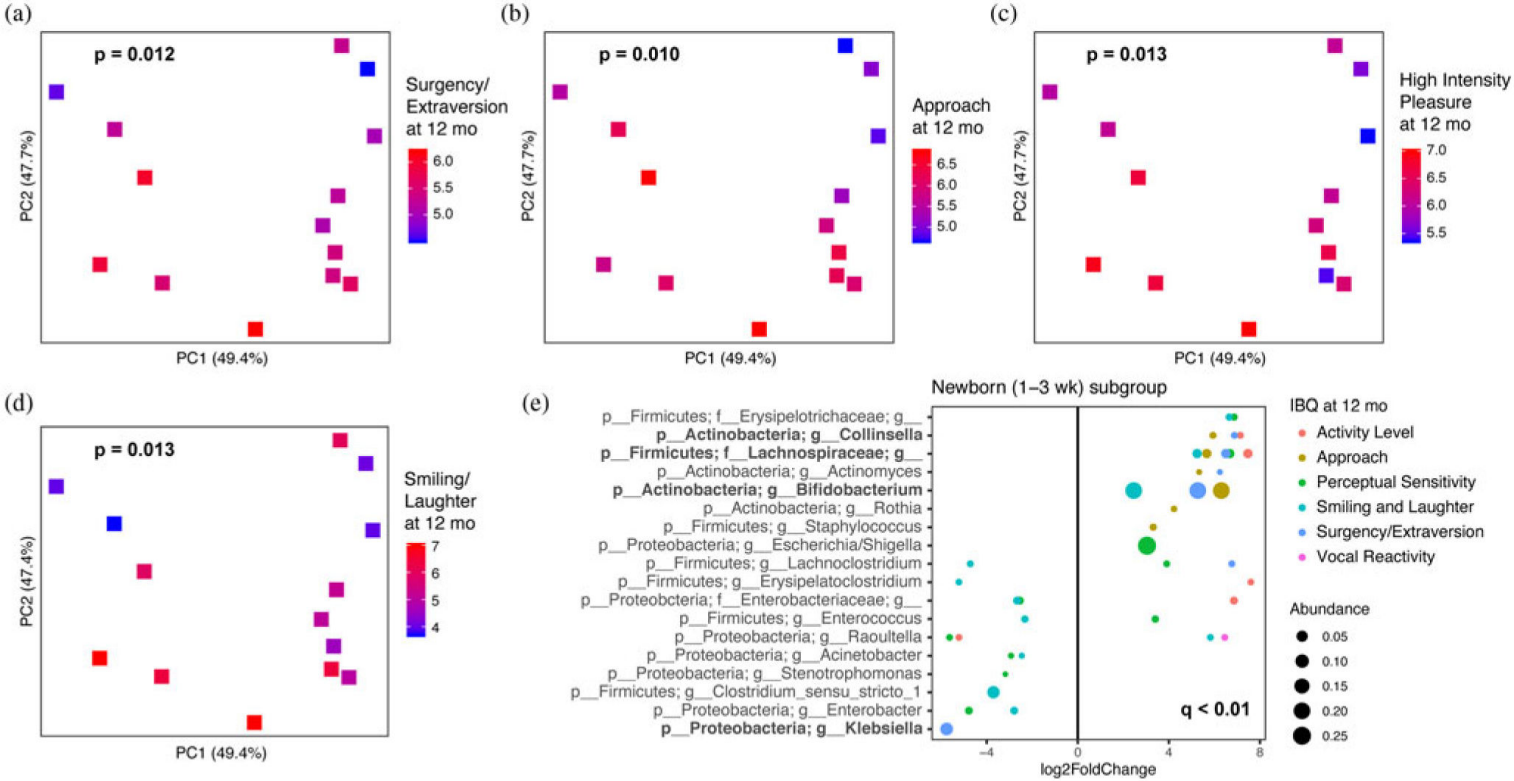
Microbial beta diversity of newborn (1–3 weeks old) fecal microbiota associated with Infant Behavior Questionnaire-Revised (IBQ-R) scale for (a) surgency/extraversion and its subscales, including (b) approach, (c) high-intensity pleasure, and (d) smiling/laughter at age 12 months. Corresponding IBQ-R scores are represented by color gradient. *P* values for these associations were determined using PERMANOVA (permutational multivariate analysis of variance) after adjusting for infant sex. (e) Specific genera from newborn (1–3 weeks old) fecal microbiota were significantly (*q* < 0.1) associated with surgency/extraversion, activity level, approach, perceptual sensitivity, smiling/laughter, and vocal reactivity subscales at age 12 months using DESeq2 model with infant sex as a covariate. Log_2_ fold change is used to show the effect size and direction of these associations. Dot size is proportional to the mean relative abundance of the genus across all samples and color corresponds to the IBQ-R scales.

**Figure 3.**
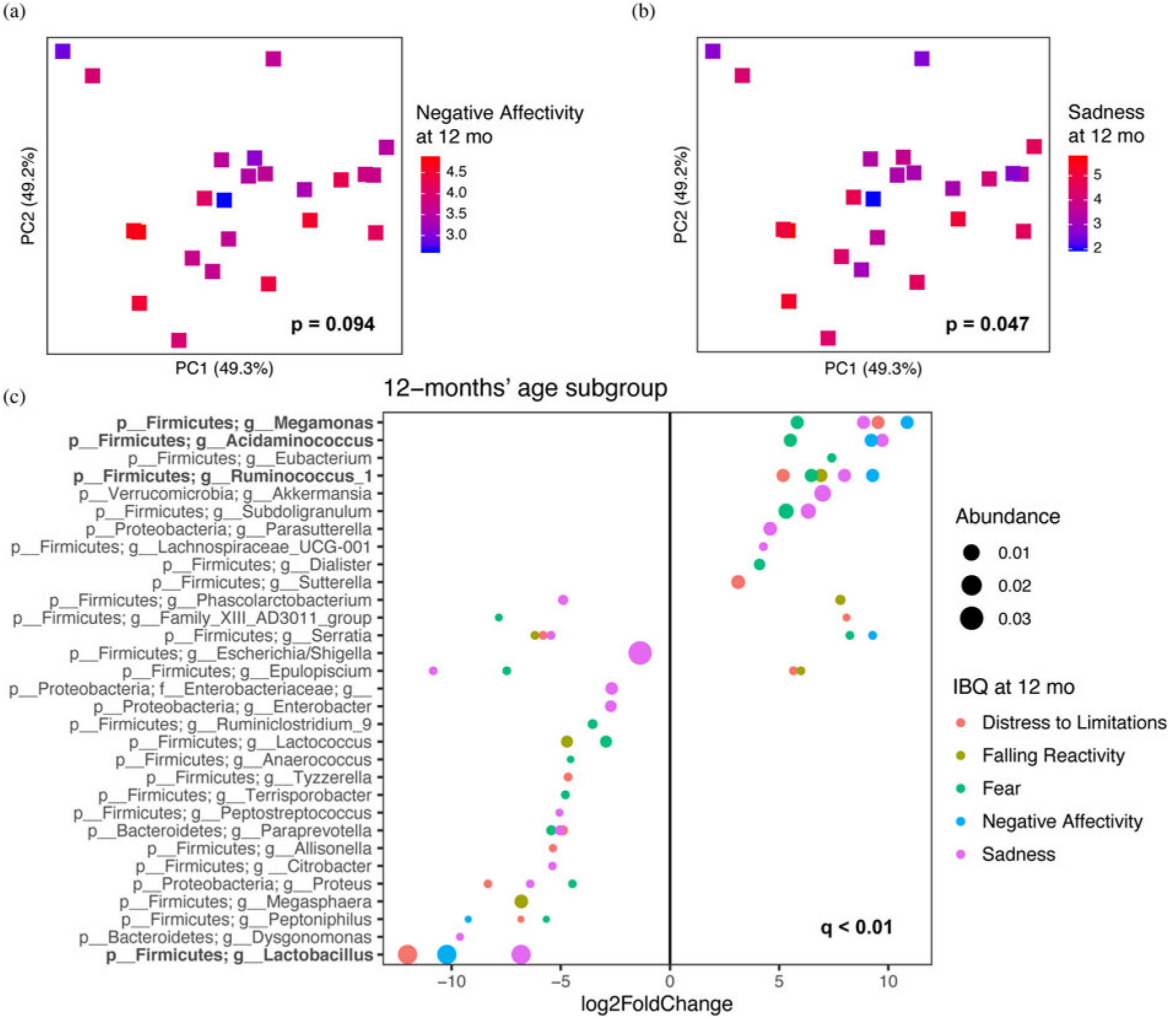
Microbial beta diversity of 12-month-old infant fecal microbiota associated with Infant Behavior Questionnaire-Revised (IBQ-R) scale for negative affectivity (a) and its component subscale sadness (b) at age 12 months. Corresponding IBQ-R scores are represented by color gradient. *P* values for these associations were determined using PERMANOVA (permutational multivariate analysis of variance) after adjusting for infant sex and duration of breastfeeding. (c) Specific genera from fecal microbiota at 12 months of age were significantly (*q* < 0.1) associated with negative affectivity, distress to limitations, falling reactivity, fear, and sadness scales at age 12 months using DESeq2 with infant sex and duration breastfeeding as covariates. Log_2_ fold change is used to show the effect size and direction of these associations. Dot size is proportional to the mean relative abundance of the genus and color corresponds to the IBQ-R scales.

We investigated the relationship between gut microbiota at each age group and IBQ-R scores at 12-months of age (Table 1 and Supplementary Material Table S2) by alpha diversity (Supplementary Material Table S3) and beta diversity measures (Table 2). We found a trend toward the Shannon index of fecal microbiota at age 2 months being negatively associated with negative affectivity score at 12 months of age, though this was not statistically significant ((β = −0.57, *p* = .06). No other IBQ-R domain or subscale at age 12 months demonstrated a significant association with the microbial alpha diversity measures at different ages. We found that gut microbial beta diversity at age 1–3 weeks was associated with surgency/extraversion (*R*^2^ = 0.276, *p* = .012) as well as its subscales, including approach (*R*^2^ = 0.285, *p* = .010), high-intensity pleasure (*R*^2^ = 0.275, *p* = .013), and smiling/laughter (*R*^2^ = 0.273, *p* = .013) (Figures 2a-2d). In addition, we found a trend toward an association between concurrent gut microbial beta diversity with negative affectivity (*R*^2^ = 0.101, *p* = .094), although it did not reach statistical significance, and an association with its sadness subscale (*R*^2^ = 0.126, *p* = .047) at 12 months of age (Figures 3a and 3b).

### Individual taxa associated with temperament

Based on the relationships between microbiota beta diversity at age 1–3 weeks and IBQ-R scores at age 12 months, we then performed differential abundance testing to identify specific genera from the infant gut microbiota that are associated with the corresponding IBQ-R scores at age 12 months. Of note, genus *Bifidobacterium*, an unclassified *Lachnospiraceae*, and genus *Collinsella* were positively associated with the surgency/extraversion scale as well as two or more of its subscales (activity level, approach, smiling/laughter, or perceptual sensitivity) at age 12 months (Figure 2e). In addition, there was a negative association between genus *Klebsiella* in the microbiota at age 1–3 weeks and the surgency/extraversion scores at age 12 months (Figure 2e). Although there was a significant association between beta diversity at age 1–3 weeks and high-intensity pleasure scores at 12 months (Figure 2c; *p* = .013), no individual taxa were associated with this IBQ-R subscale.

Next, we investigated whether any individual taxa in the concurrent microbiome were associated with the negative affectivity domain score and its subscales at age 12 months. This analysis revealed genera *Megamonas, Acidaminococcus* and *Ruminococcus-1* to be positively associated with negative affectivity and two or more of its subscales (sadness, distress to limitations, falling reactivity, and fear) at age 12 months (Figure 3c). In addition, negative affectivity and its subscales sadness and distress to limitations showed significant negative associations with genus *Lactobacillus* (Figure 3c).

## Discussion

We found that intestinal microbial composition and diversity of infants at 1–3 weeks, 2 months, and 12 months of age were associated with two temperament domains – surgency/extraversion and negative affectivity – at age 12 months. This study adds to the growing literature demonstrating associations between gut microbiome composition and temperament in infancy. While exploratory, our results also suggest the potential existence of sensitive periods during which the coinciding maturation of the gut microbiome and brain may have an influence on infant neurodevelopmental outcomes.

### Gut microbiome diversity and temperament

Previous studies have shown that the composition of the gut microbiome in early infancy is associated with individual differences in temperament in late infancy and early childhood that are predictive of risk for affective disorders in childhood and adulthood. We found that alpha diversity and beta diversity in early infancy were associated with IBQ-R scores at age 12 months. Alpha diversity at age 2 months was inversely associated with negative affectivity scores at age 12 months, although it did not reach the standard criteria for statistical significance (*p* = .06). This is consistent with another study that reported negative associations between gut microbiome alpha diversity at age 2.5 months and negative affectivity scores at age 6 months (Aatsinki et al., 2019). The significant association between beta diversity and surgency/extraversion observed in this study is also consistent with previous work. A study of gut microbiome composition and temperament of toddlers similarly detected a positive relationship between phylogenetic diversity (another alpha diversity measure) and surgency/extraversion (Christian et al., 2015).

Taken together, our results suggest that gut microbial diversity in early infancy predicts temperament traits related to negative affectivity and surgency/extraversion, which have been elsewhere related to lifetime risk for developing affective disorders. Negative affectivity during infancy is associated with risk for developing depressive and anxiety symptoms later in life (Abulizi et al., 2017; Compas et al., 2004; De Pauw & Mervielde, 2010). Surgency/extraversion in infancy has shown mixed associations with mental health outcomes later in life. For example, it is associated with greater self-regulation in childhood and lower risk for depressive symptoms (Komsi et al., 2006; Rothbart & Posner, 2015). However, surgency/extraversion scores in infancy have also been associated with adverse outcomes such as negative peer behaviors and externalizing problems in childhood and adolescence (Berdan, Keane, & Calkins, 2008; Dollar & Stifter, 2012; Honomichl & Donnellan, 2012). We are aware of only one longitudinal study with multiple assessments of infant gut microbiome composition and child outcomes, which reported no associations between alpha or beta diversity at 1, 6, or 12 months of age with behavioral problems, measured using the Childhood Behavioral Checklist at age 2 (Loughman, Ponsonby, et al., 2020). This suggests that the association between gut microbial alpha and beta diversity in early infancy and behavioral outcomes may be attenuated by 2 years of age. More longitudinal studies are needed in this area as questions about the role of microbial diversity in shaping neurodevelopmental outcomes, such as temperament, remain unanswered.

### Microbial taxa and temperament

We replicated several associations between microbial composition and temperament reported in previous studies. We found a positive association between *Bifidobacterium* at 1–3 weeks and surgency/extraversion scores. *Bifidobacterium* is an essential group of bacteria that digest human milk oligosaccharides that are otherwise indigestible (Gnoth, Kunz, Kinne-Saffran, & Rudloff, 2000; Sela & Mills, 2010). Human milk oligosaccharides are believed to regulate development of the gut microbiome by promoting the growth of beneficial bacteria and preventing pathogenic bacteria from colonizing the infant gut (Liévin et al., 2000). *Bifidobacterium* appears to be an important predictor of infant temperament, as a similar study also detected a significant positive association between *Bifidobacterium* at age 2.5 months and surgency/extraversion scores at age 6 months (Aatsinki et al., 2019). Our results suggest that the presence of *Bifidobacterium* may be important as early as 1–3 weeks after birth. Such results point to the potential significance of this genus in the development of extraversion, given the relationship between surgency/extraversion scores in infancy and surgency later in childhood (Komsi et al., 2006). A study in toddlers reported that surgency scores were associated, for boys only, with different genera from those observed in this study (*Parabacteroides*, *Dialister*, and *Rikenellaceae*) (Christian et al., 2015).

We also found associations between several genera at age 1–3 weeks and 12 months with surgency/extraversion. We found a positive association with an unclassified *Lachnospiraceae* and a negative association with *Klebsiella*. The associations with *Lachnospiraceae* are generally consistent with results from a longitudinal study that reported positive associations between this bacteria at age 12 months and internalizing problems in 2-year-old children (Loughman, Ponsonby, et al., 2020). The negative associations between *Klebsiella* and surgency/extraversion may not be surprising as *Klebsiella* is pathogenic and thought to be a gas-producing bacteria that may cause intestinal discomfort (Savino et al., 2011). *Klebsiella* levels have been implicated in infant colic and infants with colic have low emotional regulation (Loughman, Quinn, et al., 2020; Savino et al., 2017; Stifter & Spinrad, 2002).

We found associations between several concurrent microbial taxa and negative affectivity scores at age 12 months. This included a positive association with *Ruminococcus-1*, consistent with previously observed associations of the *Ruminococcaceae* family with depressive symptoms in adults (Valles-Colomer et al., 2019). We also found a negative association between *Lactobacillus* and negative affectivity, which is consistent with the extant literature. Supplementation with probiotics containing *Lactobacillus* strains has been shown to reduce crying behaviors of infants with colic (Sung et al., 2018) and symptoms of anxiety and depression in adults (Messaoudi et al., 2011).

The mechanisms by which specific microbial taxa may influence behavior remain unclear. However, mouse models offer potential mechanisms: gut microbial composition was demonstrated to influence neurological biomechanisms such as the expression of neurotransmitters and their hormones, including dopamine and serotonin (Clarke et al., 2013; Diaz Heijtz et al., 2011; Neufeld, Kang, Bienenstock, & Foster, 2011). Another potential mechanism is differences in neuronal survival and neurogenesis in regions such as the striatum, amygdala, and hippocampus (Diaz Heijtz et al., 2011; Ogbonnaya et al., 2015; Stilling et al., 2015). The gut microbiome may also influence brain function by regulating microglial activity (Erny et al., 2015).

### Sensitive periods in gut microbiome and neurodevelopment

The sensitive periods during which the gut microbiome may influence neurodevelopment and behavior are unknown. A growing body of work suggests that infancy may be a sensitive period in which the microbiome may have far-reaching influence on later neuropsychological health and behavior (Borre et al., 2014; Jašarević et al., 2016; Stilling et al., 2014), although some scholars have argued that it may extend into the first 1,000 days of life or even into adolescence (Cowan, Dinan, & Cryan, 2020; Robertson, Manges, Finlay, & Prendergast, 2019). Early life may be a particularly important period due to founder species that may influence the long-term composition of the gut microbiome (Litvak & Bäumler, 2019). The first year of life is a dynamic period of brain development involving the formation of functional networks (Gao, Lin, Grewen, & Gilmore, 2017; Knickmeyer et al., 2008). Infants exhibit rapid social and emotional development, including the emergence of temperament. The early postnatal phase is one of heightened plasticity, and perturbations to the gut and brain may have far-reaching consequences for mental health risk over the life course. Our results, and those of previous studies, provide preliminary evidence to support infancy as a sensitive period during which gut microbiome composition may impact neurodevelopmental outcomes (Aatsinki et al., 2019; Carlson et al., 2018; Christian et al., 2015). The importance of these overlapping windows of development are increasingly recognized as scholars have hypothesized that the microbiome may be a mechanism in the developmental origins of health and disease (Stiemsma & Michels, 2018; Stinson, 2020). Mouse models suggest that early life stages are sensitive periods in which the microbiome influences neurodevelopment. The maternal gut microbiome may regulate fetal brain development *in utero* through the production of microbial metabolites that promote axonogenesis (Vuong et al., 2020). Another pathway is through transmission of dysbiosis from mothers to pups, which disrupts social and behavioral developmental processes (Buffington et al., 2016; Champagne-Jorgensen et al., 2020). Evidence suggests that weaning and adolescence are transitions marked by microbial instability and these are thus other likely sensitive periods for the development of the microbiome–gut–brain axis (Cowan et al., 2020). Additional studies are needed to elucidate the number and duration of overlapping sensitive periods of gut microbiome and neurodevelopment.

### Limitations

The strengths of this study include a longitudinal design of stool sampling at multiple timepoints across infancy, including two timepoints earlier than any previous study. However, the results should be considered in the light of several limitations.

First, a relatively small sample size was used in this study. While such sample sizes are common in human microbiome research, they limit statistical power to detect associations with small, but meaningful, effect sizes. Future studies should be conducted with larger samples and consideration of more potential covariates. Second, we assessed infant temperament by maternal report. While parents are able to best observe infants across a wide variety of environments (Gartstein & Rothbart, 2003), parental reports do not always correlate strongly with observer reports in standardized conditions (Mangelsdorf, Schoppe, & Buur, 2000). Third, microbiome analysis was performed using 16S ribosomal RNA gene sequencing, which provides information on microbial composition but not function (e.g., bacterial gene content or metabolite levels). Fourth, we conducted a large number of comparisons and, importantly, acknowledge the possibility that this may have led to spurious findings. However, some concern over the number of comparisons is ameliorated by the fact that many of the associations we detected are consistent with previous studies. Fifth, with the small sample size we were not able to assess other variables that may influence infant gut microbiome composition and we had to be discerning in the selection of covariates in order to preserve statistical power. Sixth, we were unable to prove the existence of sensitive periods because we could not compare influences at various timepoints on phenotypes later in life than 12 months of age. Additional longitudinal studies are needed to identify specific critical windows for contributions of the gut microbiome to inter-individual differences in behavioral and mental health outcomes.

## Conclusions

Our study shows that composition of the gut microbiome at 1–3 weeks, 2 months, and 12 months of age is associated with infant temperament at age 12 months. This suggests that early infancy may be a sensitive period for gut microbiome and brain crosstalk. Our results may inform early life interventions, such as probiotics that target the gut microbiome to promote optimal infant development. While this study supports the hypothesis that the gut microbiome in early life has far-reaching consequences for neuro-development, further studies are needed to understand the mechanisms involved in this process.

## Supplementary Material

Supplementary Material

## Figures and Tables

**Table 1.** Characteristics of the study subjects

Measure	Total (*N* = 67)	Child age			
		1–3 weeks (*N* = 23)	2 months (*N* = 25)	6 months (*N* = 16)	12 months (*N* = 27)
Female, *N*	32/67 (48%)	11/23 (48%)	11/25 (44%)	8/16 (50%)	17/27 (63%)
Cesarean section^a^, *N*	19/67 (28%)	5/23 (22%)	3/25 (12%)	9/16 (56%)	9/27 (33%)
Birthweight (kg): mean ± *SD*	3.3 ± 0.5 (one missing value)	3.3 ± 0.5	3.4 ± 0.3 (one missing value)	3.4 ± 0.6	3.1 ± 0.5
Birth length (cm): mean ± *SD*	50.0 ± 2.5 (six missing values)	50.3 ± 2.3 (one missing value)	50.1 ± 2.3 (five missing values)	50.7 ± 2.5 (one missing value)	49.3 ± 2.6
Breastfeeding duration (months): mean ± *SD*	5.9 ± 4.7	5.3 ± 4.6	6.3 ± 4.5	6.4 ± 4.6	6.4 ± 4.7
Antibiotic or antifungal use, *N*	26/67 (63%)	0/23 (0%)	3/25 (12%)	4/16 (25%)	10/27 (37%)
Mother’s race, *N*					
White^b^	21/67 (31%)	5/23 (22%)	7/25 (28%)	4/16 (25%)	9/27 (33%)
African American^c^	2/67 (3%)	0/23 (0%)	1/25 (5%)	1/16 (6%)	1/27 (4%)
Asian	8/67 (12%)	6/23 (26%)	3/25 (10%)	0/16 (0%)	4/27 (15%)
Multi-ethnic	6/67 (9%)	1/23 (4%)	3/25 (14%)	4/16 (25%)	2/27 (7%)
Hispanic or Latino	30/67 (45%)	11/23 (48%)	11/25 (43%)	7/16 (44%)	11/27 (41%)
Mother’s parity^d^: mean ± *SD*	0.8 ± 0.9	0.7 ± 0.8	0.7 ± 0.9	0.9 ± 0.6	1.0 ± 1.0
Surgency/extraversion factor: mean ± *SD*	5.3 ± 0.5	5.4 ± 0.5	5.1 ± 0.5	5.3 ± 0.6	5.3 ± 0.6
Negative affectivity factor: mean ± *SD*	3.6 ± 0.6	3.7 ± 0.6	3.3 ± 0.6	3.5 ± 0.6	3.8 ± 0.6
Orienting/regulation factor: mean ± *SD*	4.8 ± 0.5	5.0 ± 0.5	4.7 ± 0.5	4.8 ± 0.5	4.7 ± 0.5

a Delivery types for non-Cesarean section include normal spontaneous vaginal delivery, vaginal birth after cesarean, outlet or low forceps, outlet or low vacuum, mid forceps, and unknown.

b White, European, North African, Middle Eastern.

c African American or Black.

d Pregnancy history counts before current pregnancy.

**Table 2.** Beta diversity association with Infant Behavior Questionnaire-Revised (IBQ-R) scores at 12 months of age using PERMANOVA (permutational multivariate analysis of variance), adjusted for infant sex and breastfeeding duration (for analyses of the 1–3 weeks subgroup, only infant sex was adjusted due to lack of breastfeeding variability)

Infant age at sample	IBQ-R domain and subscales	*R* ^2^	*p* value
1–3 weeks			
	Negative affectivity	0.004	0.975
	Surgency/extraversion	0.276	0.012*^a^
	Activity level	0.038	0.667
	Approach	0.285	0.010*
	High-intensity pleasure	0.275	0.013*
	Perceptual sensitivity	0.088	0.360
	Smiling/laughter	0.273	0.013*
	Vocal reactivity	0.029	0.739
	Orienting/regulation	0.089	0.346
2 months			
	Negative affectivity	0.006	0.927
	Surgency/extraversion	0.080	0.256
	Orienting/regulation	0.017	0.782
6 months			
	Negative affectivity	0.012	0.897
	Surgency/extraversion	0.060	0.461
	Orienting/regulation	0.089	0.280
12 months			
	Negative affectivity	0.101	0.094
	Sadness	0.126	0.047*
	Distress to limitations	0.002	0.983
	Fear	0.047	0.356
	Falling reactivity/Rate of recovery from distress	0.097	0.102
	Surgency/extraversion	0.007	0.884
	Orienting/regulation	0.007	0.870

a* indicates a *p* value ≤ 0.05.
