# Supplementary material for: Development of the infant gut microbiome predicts temperament across the first year of life
Source: Dev Psychopathol. Author manuscript; Available in PMC 2022 Dec 10. (PMC9463039; doi:10.1017/S0954579421000456)
Supplement: Supplementary Material [file NIHMS1833066-supplement-Supplementary_Material.docx]

**Supplementary Figure 1.** Notched box plots showing changes in relative abundance of phyla Firmicutes (left) and Proteobacteria (right) at different ages during the first year of life representing 91 samples collected from 67 infant donors. Each dot represents a sample. P-value for relative abundance changes by age is determined by one-way ANOVA after adjusting for subject. 95% confidence interval around the median is displayed by the notch.


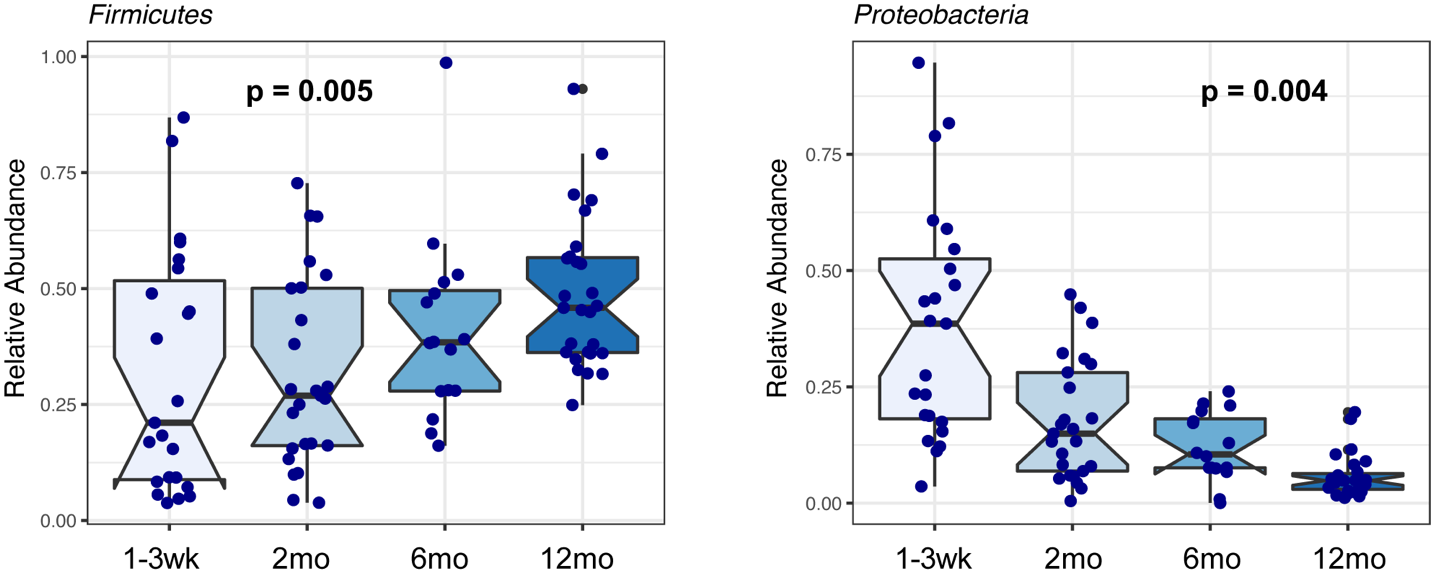


**Supplementary Table 1. Covariate selection algorithm based on univariable model of all independent variables associated with beta diversity at different ages using PERMANOVA.**

| **Sample subgroup** | **Independent variables** | **R^2** | **p-value** |
| --- | --- | --- | --- |
| Total | Age + Infant sex (M/F) | 0.005 | 0.599 |
| 1-3 weeks | Infant sex (M/F) | 0.051 | 0.337 |
| 2 months | Infant sex (M/F) | 0.126 | 0.04* |
| 6 months | Infant sex (M/F) | 0.057 | 0.456 |
| 12 months | Infant sex (M/F) | 0.043 | 0.346 |
| Total | Age + Breastfeeding duration | 0.032 | 0.026* |
| 1-3 weeks | Breastfeeding duration^a^ | N/A | N/A |
| 2 months | Breastfeeding duration | 0.021 | 0.644 |
| 6 months | Breastfeeding duration | 0.067 | 0.388 |
| 12 months | Breastfeeding duration | 0.031 | 0.465 |
| Total | Age + c-section (Y/N) | 0.003 | 0.763 |
| 1-3 weeks | c-section (Y/N) | 0.022 | 0.671 |
| 2 months | c-section (Y/N) | 0.034 | 0.468 |
| 6 months | c-section (Y/N) | 0.024 | 0.754 |
| 12 months | c-section (Y/N) | 0.053 | 0.257 |
| Total | Age + Antibiotics or antifungals (Y/N) | 0.006 | 0.560 |
| 1-3 weeks | Antibiotics or antifungals (Y/N) | 0.012 | 0.829 |
| 2 months | Antibiotics or antifungals (Y/N) | 0.053 | 0.300 |
| 6 months | Antibiotics or antifungals (Y/N) | 0.110 | 0.201 |
| 12 months | Antibiotics or antifungals (Y/N) | 0.015 | 0.702 |

^a^For 1-3 weeks subgroup analyses, only infant sex was adjusted for due to lack of breastfeeding variability.

**Supplementary Table 2. Summary of the IBQ subscale scores at 12 months of age represented by mean and standard deviation.**

| **12 months IBQ scale** | **Total** | **1-3 weeks** | **2 months** | **6 months** | **12 months** |
| --- | --- | --- | --- | --- | --- |
| Surgency/Extraversion factor | 5.3 +/- 0.5 | 5.4 +/- 0.5 | 5.1 +/- 0.5 | 5.3 +/- 0.6 | 5.3 +/- 0.6 |
| Activity Level subscale | 4.6 +/- 0.8 | 4.7 +/- 0.8 | 4.4 +/- 0.8 | 4.6 +/- 0.7 | 4.7 +/- 0.9 |
| Smiling and Laughter subscale | 5.3 +/- 1.0 | 5.3 +/- 1.1 | 5.1 +/- 1.0 | 5.3 +/- 1.0 | 5.3 +/- 0.9 |
| High Intensity Pleasure subscale | 6.1 +/- 0.6 | 6.2 +/- 0.5 | 5.9 +/- 0.7 | 6.0 +/- 0.7 | 6.1 +/- 0.8 |
| Approach subscale | 5.9 +/- 0.7 | 5.8 +/- 0.7 | 5.8 +/- 0.5 | 6.1 +/- 0.7 | 5.8 +/- 0.6 |
| Perceptual Sensitivity subscale | 4.5 +/- 1.1 | 4.8 +/- 1.0 | 4.0 +/- 0.9 | 4.4 +/- 1.2 | 4.4 +/- 1.1 |
| Vocal Reactivity subscale | 5.6 +/- 0.8 | 5.4 +/- 0.8 | 5.4 +/- 0.8 | 5.7 +/- 0.8 | 5.6 +/- 0.8 |
| Negative Affectivity factor | 3.6 +/- 0.6 | 3.7 +/- 0.6 | 3.3 +/- 0.6 | 3.5 +/- 0.6 | 3.8 +/- 0.6 |
| Distress to Limitations subscale | 4.2 +/- 0.8 | 4.2 +/- 0.6 | 4.0 +/- 0.7 | 4.0 +/- 0.8 | 4.4 +/- 0.9 |
| Fear subscale | 3.5 +/- 1.0 | 3.6 +/- 1.0 | 3.3 +/- 0.9 | 3.5 +/- 1.0 | 3.8 +/- 0.9 |
| Falling Reactivity/Rate of Recovery from Distress subscale | 4.9 +/- 0.7 | 4.8 +/- 0.5 | 5.0 +/- 0.9 | 5.1 +/- 0.5 | 4.8 +/- 0.7 |
| Sadness subscale | 3.6 +/- 1.0 | 3.5 +/- 0.9 | 2.9 +/- 0.8 | 3.6 +/- 1.1 | 3.9 +/- 1.0 |
| Orienting/Regulation factor | 4.8 +/- 0.5 | 5.0 +/- 0.5 | 4.7 +/- 0.5 | 4.8 +/- 0.5 | 4.7 +/- 0.5 |
| Duration of Orienting subscale | 4.2 +/- 0.9 | 4.4 +/- 0.9 | 3.8 +/- 0.9 | 4.5 +/- 0.8 | 4.1 +/- 0.9 |
| Low Intensity Pleasure subscale | 5.1 +/- 0.9 | 5.4 +/- 0.7 | 5.0 +/- 0.9 | 5.1 +/- 0.9 | 4.9 +/- 0.9 |
| Soothability subscale | 4.8 +/- 0.7 | 5.0 +/- 0.8 | 4.8 +/- 0.8 | 4.7 +/- 0.5 | 4.8 +/- 0.8 |
| Cuddliness subscale | 5.1 +/- 0.8 | 5.3 +/- 0.8 | 5.2 +/- 0.9 | 5.0 +/- 0.5 | 4.9 +/- 0.8 |

**Supplementary Table 3. Alpha diversity association with IBQ scores at 12 months of age using multivariate linear regression models adjusting for infant sex and breastfeeding duration^a^.**

| **Sample subgroup** | **α-diversity measure** | **IBQ scale at 12 mo** | **Beta coeff** | **p-value** | **Model R^2** | **Model**  **F-value and DF** | **Model**  **p-value** |
| --- | --- | --- | --- | --- | --- | --- | --- |
| 1-3 weeks | Chao1 | Negative Affectivity | 19.8 | 0.64 | 0.032 | F(2,11)=0.18 | 0.84 |
| 1-3 weeks | Chao1 | Surgency/Extraversion | -30.7 | 0.54 | 0.046 | F(2,11)=0.26 | 0.77 |
| 1-3 weeks | Chao1 | Orienting/Regulation | -17.4 | 0.71 | 0.024 | F(2,11)=0.13 | 0.88 |
| 2 months | Chao1 | Negative Affectivity | -54.0 | 0.22 | 0.297 | F(3,13)=1.83 | 0.19 |
| 2 months | Chao1 | Surgency/Extraversion | 41.9 | 0.48 | 0.238 | F(3,13)=1.35 | 0.30 |
| 2 months | Chao1 | Orienting/Regulation | 42.6 | 0.41 | 0.249 | F(3,13)=1.43 | 0.28 |
| 6 months | Chao1 | Negative Affectivity | -64.6 | 0.58 | 0.108 | F(3,8)=0.32 | 0.81 |
| 6 months | Chao1 | Surgency/Extraversion | -88.9 | 0.36 | 0.169 | F(3,8)=0.54 | 0.67 |
| 6 months | Chao1 | Orienting/Regulation | -136.7 | 0.26 | 0.213 | F(3,8)=0.72 | 0.57 |
| 12 months | Chao1 | Negative Affectivity | 79.6 | 0.32 | 0.076 | F(3,20)=0.55 | 0.66 |
| 12 months | Chao1 | Surgency/Extraversion | -0.53 | 0.99 | 0.028 | F(3,20)=0.19 | 0.90 |
| 12 months | Chao1 | Orienting/Regulation | 57.0 | 0.46 | 0.055 | F(3,20)=0.39 | 0.76 |
| 1-3 weeks | Shannon | Negative Affectivity | 0.26 | 0.41 | 0.063 | F(2,11)=0.37 | 0.70 |
| 1-3 weeks | Shannon | Surgency/Extraversion | -0.44 | 0.25 | 0.118 | F(2,11)=0.73 | 0.50 |
| 1-3 weeks | Shannon | Orienting/Regulation | -0.40 | 0.27 | 0.109 | F(2,11)=0.67 | 0.53 |
| 2 months | Shannon | Negative Affectivity | -0.57 | *0.06* | 0.369 | F(3,13)=2.54 | 0.10 |
| 2 months | Shannon | Surgency/Extraversion | 0.28 | 0.50 | 0.193 | F(3,13)=1.03 | 0.41 |
| 2 months | Shannon | Orienting/Regulation | 0.33 | 0.37 | 0.214 | F(3,13)=1.18 | 0.35 |
| 6 months | Shannon | Negative Affectivity | -0.05 | 0.92 | 0.120 | F(3,8)=0.36 | 0.78 |
| 6 months | Shannon | Surgency/Extraversion | -0.27 | 0.55 | 0.160 | F(3,8)=0.51 | 0.69 |
| 6 months | Shannon | Orienting/Regulation | -0.60 | 0.28 | 0.246 | F(3,8)=0.87 | 0.50 |
| 12 months | Shannon | Negative Affectivity | 0.30 | 0.27 | 0.134 | F(3,20)=1.03 | 0.40 |
| 12 months | Shannon | Surgency/Extraversion | -0.07 | 0.77 | 0.082 | F(3,20)=0.60 | 0.62 |
| 12 months | Shannon | Orienting/Regulation | 0.08 | 0.75 | 0.083 | F(3,20)=0.60 | 0.62 |

^a^For 1-3 weeks subgroup analyses, only infant sex was adjusted for due to lack of breastfeeding variability.
